# Functional Redundancy of Cyclase-Associated Proteins CAP1 and CAP2 in Differentiating Neurons

**DOI:** 10.3390/cells10061525

**Published:** 2021-06-17

**Authors:** Felix Schneider, Isabell Metz, Sharof Khudayberdiev, Marco B. Rust

**Affiliations:** 1 Molecular Neurobiology Group, Institute of Physiological Chemistry, University of Marburg, 35032 Marburg, Germany; felix.schneider@staff.Uni-Marburg.DE (F.S.); isabell.metz@uni-marburg.de (I.M.); khudaybe@staff.uni-marburg.de (S.K.); 2 Center for Mind, Brain and Behavior (CMBB), University of Marburg, Justus-Liebig-University Giessen, Hans-Meerwein-Strasse 6, 35032 Marburg, Germany; 3 DFG Research Training Group, Membrane Plasticity in Tissue Development and Remodeling, GRK 2213, University of Marburg, 35032 Marburg, Germany

**Keywords:** cyclase-associated protein, CAP2, CAP1, SRV2, growth cone, actin dynamics, F-actin

## Abstract

Cyclase-associated proteins (CAPs) are evolutionary-conserved actin-binding proteins with crucial functions in regulating actin dynamics, the spatiotemporally controlled assembly and disassembly of actin filaments (F-actin). Mammals possess two family members (CAP1 and CAP2) with different expression patterns. Unlike most other tissues, both CAPs are expressed in the brain and present in hippocampal neurons. We recently reported crucial roles for CAP1 in growth cone function, neuron differentiation, and neuron connectivity in the mouse brain. Instead, CAP2 controls dendritic spine morphology and synaptic plasticity, and its dysregulation contributes to Alzheimer’s disease pathology. These findings are in line with a model in which CAP1 controls important aspects during neuron differentiation, while CAP2 is relevant in differentiated neurons. We here report CAP2 expression during neuron differentiation and its enrichment in growth cones. We therefore hypothesized that CAP2 is relevant not only in excitatory synapses, but also in differentiating neurons. However, CAP2 inactivation neither impaired growth cone morphology and motility nor neuron differentiation. Moreover, CAP2 mutant mice did not display any obvious changes in brain anatomy. Hence, differently from CAP1, CAP2 was dispensable for neuron differentiation and brain development. Interestingly, overexpression of CAP2 rescued not only growth cone size in CAP1-deficient neurons, but also their morphology and differentiation. Our data provide evidence for functional redundancy of CAP1 and CAP2 in differentiating neurons, and they suggest compensatory mechanisms in single mutant neurons.

## 1. Introduction 

Mammalian cyclase-associated protein (CAP) and its yeast homolog suppressor of RAS2-V19 (SRV2, collectively referred to CAP in this manuscript) were both recognized, two decades ago, as actin-binding proteins (ABPs) [1,2], but their molecular functions remained largely unknown until recently. By exploiting recombinant proteins and mutant yeast strains, studies of the past few years implicated CAP in various steps of the actin treadmilling mechanism, thereby identifying it as a crucial regulator of actin dynamics [3,4,5,6,7,8]. Specifically, these studies revealed a cooperation with the actin-depolymerizing protein ADF/cofilin in the dissociation of actin subunits from filamentous actin (F-actin) and, hence, in F-actin disassembly [5,7,9]. Moreover, they revealed a role for CAP in nucleotide exchange on globular actin monomers (G-actin) relevant for G-actin recycling and F-actin assembly [4], and they reported an inhibitory function towards the F-actin assembly factor, inverted formin 2 (INF2) [6,8]. While these studies significantly advanced our knowledge of CAP’s molecular activities, little is known about the cellular and physiological functions. Unlike lower eukaryotes and most invertebrate species that possess a single CAP homolog, vertebrates express two closely related family members, CAP1 and CAP2, with different expression pattern [10,11]. In mice, CAP1 is present in most tissues except skeletal muscles, while CAP2 expression is restricted to a few tissues including skeletal muscle and the heart [10]. These findings led to the assumption that both proteins acquired cell-type-specific functions with CAP2 being the dominant family member in striated muscles [2]. Indeed, recent studies identified CAP2 as a crucial regulator of myofibril differentiation in both skeletal and cardiac muscles [12,13], and they reported a cardiomyopathy associated with dilated ventricles and impaired heart physiology as well as impaired skeletal muscle development for systemic CAP2 knockout (CAP2-KO) mice [12,14,15,16,17]. Instead, systemic CAP1-KO mice died from unknown causes during embryonic development [18]. Differently from most other tissues, CAP1 and CAP2 are both expressed in the brain [10,11], and recent studies unraveled important functions for both in hippocampal neurons. Specifically, shRNA-mediated knockdown in isolated rat hippocampal neurons revealed a role for CAP2 in the morphology and function of dendritic spines [19], F-actin-enriched dendritic protrusions forming the postsynaptic compartment of most excitatory synapses in the brain [20,21]. Moreover, CAP2 dysregulation has been implicated in synaptic defects of Alzheimer’s disease [19]. Instead, hippocampal neurons from brain-specific CAP1-KO mice displayed an altered morphology and function of growth cones [9], dynamic F-actin-rich structures at the tip of neurites that sense environmental guidance cues and navigate axons through the developing brain to their target regions [22]. Consequently, neuron connectivity was compromised in CAP1-KO brains [9]. These findings are in line with a model in which CAP1 controls actin-dependent mechanisms during neuron differentiation, while CAP2 is relevant for actin regulation in differentiated neurons. However, the function of CAP2 during differentiation of hippocampal neurons has not been studied to date. 

In the present study, we found CAP2 expressed during differentiation and abundant in growth cones from hippocampal neurons. We therefore hypothesized that CAP2 plays a crucial role in growth cones, similar to CAP1. Unlike CAP1-KO neurons, neuron differentiation and growth cone morphology were unchanged in neurons from CAP2-KO mice, and CAP2-KO brains did not display obvious brain developmental defects. However, overexpression of CAP2 rescued growth cone size and morphology in CAP1-KO neurons and normalized their differentiation. Our data revealed functional redundancy of CAP1 and CAP2 in neurons, and they suggest compensatory mechanisms in single KO mice. 

## 2. Material and Methods

### 2.1. Transgenic Mice

CAP2^−/−^ mice were generated by breeding heterozygous CAP2 mice (CAP2^+/−^) obtained from the European Conditional Mouse Mutagenesis Program (EUCOMM). Generation of conditional CAP1 mice has been reported before [9]. CAP1-deficient hippocampal neurons were obtained from conditional CAP1 mice (CAP1^flx/flx^) additionally expressing a Cre transgene under control of the nestin promoter [23]. Mice were housed in the animal facility of Marburg University on 12 h dark–light cycles with food and water available ad libitum. Treatment of mice was in accordance with the German law for conducting animal experiments and followed the guidelines for the care and use of laboratory animals of the U.S. National Institutes of Health. Sacrificing of mice was approved by internal animal welfare authorities at Marburg University (AK-5-2014-Rust, AK-6-2014-Rust, AK-12-2020-Rust), breeding of brain-specific CAP1 mutant mice was approved by the RP Giessen (G22-2016). 

### 2.2. Cell Culture and Transfection

Primary hippocampal neurons from embryonic day 18 (E18) mice were prepared as previously described [24]. Briefly, hippocampi were dissociated individually to keep their genetic identity and seeded with a density of 31,000 neurons per cm^2^ on 0.1 mg/mL poly-l-lysine-coated coverslips. Neurons were maintained for 5 h to 3 d in a humidified incubator at 37 °C with 5% CO_2_ in neurobasal medium containing 2% B27, 2 mM GlutaMax, 100 µg/mL streptomycin, and 100 U/mL penicillin (Gibco, Invitrogen, Waltham, MA, USA). For overexpression of plasmids, neurons were nucleofected prior to plating with the Amaxa nucleofector system (Lonza, Basel, Switzerland) according to the manufacturer’s protocol. For each nucleofection, 3 µg plasmid was transfected into 250,000 neurons, which were then plated at a density of 66,000 neurons per cm^2^. For replating, neurons were seeded in wells coated with 0.05 mg/mL poly-L-lysine and detached with TrypLE™ Express (Gibco) at DIV2. A detailed protocol of the replating procedure was provided in a recent study [25]. Thereafter, neurons were pelleted at 7000 rpm for 7 min, plated on 0.1 mg/mL poly-l-lysine-coated coverslips, and fixed 24 h later. The following constructs were used: GFP-CAP1, mCherry-CAP1, and myc-CAP1 [9], GFP-CAP2 and myc-CAP2 [19], and GFP (GeneScript, Piscataway, NJ, USA). Three different shRNAs were used to knockdown CAP2: sh2 (5′-CCTTTGAGAATGAGGATAA-3′), sh3 (5′-AGAAGTGGAGAGTGGAATA-3′), and sh4 (5′-CAGATGACAAGAAGACATA-3′). 5′-AAACCTTGTGGTCCTTAGG-3′ was used as control shRNA [26]. 

### 2.3. Immunocytochemistry

Cultured neurons were fixed for 10 min in 4% paraformaldehyde (PFA) in PBS under cytoskeleton preserving conditions. After 5 min incubation with 0.4% gelatin and 0.5% Triton-X100 in PBS (carrier solution), neurons were incubated with following primary antibodies (in carrier solution): rabbit anti-doublecortin (1:500, Abcam, Cambridge, UK), rabbit anti-GFP (1:1000, ThermoFisher Scientific, Waltham, MA, USA), mouse anti-c-Myc (1:200, ThermoFisher Scientific), chicken anti-mCherry (1:500, Abcam), and mouse anti-tubulin βIII (1:200, Sigma-Aldrich, St. Louis, MO, USA). Thereafter, neurons were washed in PBS and incubated with the following secondary antibodies (in carrier solution): anti-mouse and anti-rabbit IgG coupled to either AlexaFluor488 or AlexaFluor546 (1:500, Invitrogen, Waltham, MA, USA) and AlexaFluor555-coupled anti-chicken IgG (1:500, Invitrogen). F-actin was visualized by staining with phalloidin coupled to either AlexaFluor488 (1:100, Invitrogen) or AlexaFluor647 (1:100, Cell Signaling Technologies, Danvers, MA, USA). In each experiment, neurons were stained with the DNA dye Hoechst (1:1000, Invitrogen). Image acquisition was done with a Leica TCS SP5 II confocal microscope setup, and image analysis was performed with ImageJ [27]. Briefly, growth cones were outlined by exploiting the phalloidin signal. Area and shape indices were determined by using the according measuring tools from Fiji. 

### 2.4. Growth Cone Morphology

Growth cone morphology was assessed by determining growth cone circularity (growth cone area divided by growth cone perimeter) and solidity (growth cone area divided by hull area), similar to previous studies [9]. 

### 2.5. Live Cell Imaging

For life cell imaging, neurons were seeded in a 22 mm glass-bottom dish coated with PLL as described above and cultured for 1 d. DIC imaging was done in a chamber maintained at 37 °C and the medium was exchanged with CO_2_-saturated HBS solution. Images were acquired every 5 s for 10 min at a Leica DMi8 setup. 

### 2.6. Protein Analysis

Cortices of E18.5 mice were homogenized in 500 µL RIPA buffer and cultured cells were lysed in 100µl RIPA buffer containing 50 mM Tris HCl (pH7.5), 150 mM NaCl, 0.5% NP40, 0.1% SDS, and protease inhibitor (Complete, Roche, Basel, Switzerland). After centrifugation at 14,000 rpm for 10 min at 4 °C, samples were boiled for 5 min at 95 °C in Laemmli buffer including 6% DTT. Equal protein amounts were separated by SDS PAGE and blotted onto a polyvinylidene difluoride membrane (Merck, Darmstadt, Germany) by using a wet/tank blotting system (Biorad, Hercules, CA, USA). Membranes were blocked in Tris-buffered saline (TBS) containing 5% milk powder, 0.5% Tween-20, and 0.02% sodium acid for 1 h and afterwards incubated with primary antibodies in blocking solution overnight at 4 °C. As secondary antibodies, horseradish peroxidase (HRP)-conjugated antibodies (1:10,000, Thermo Fisher Scientific, Waltham, MA, USA) or fluorescent-conjugated antibodies (1:10,000, Li-Cor Bioscience, Lincoln, NE, USA) were used and detected by chemiluminescence with ECL Plus Western blot detection system (GE Healthcare, Chicago, IL, USA) or by fluorescence with Li-Cor Odyssey imaging system. The following primary antibodies were used: mouse anti-CAP1 (1:1000, Abnova, Taoyuan City, Taiwan), mouse anti-GAPDH (1:1000, R&D System, Minneapolis, MN, USA), rabbit anti-CAP2 (1:1000), and mouse anti-α-tubulin (1:2000, Sigma-Aldrich, St. Louis, MO, USA). Signal intensities of CAP1, CAP2, and GAPDH in immunoblots were determined by using ImageStudio Light Ver 5.2. CAP1 and CAP2 levels were normalized to GAPDH. CAP2 and tubulin levels were determined by using Western blot Fiji, CAP2 level was normalized to tubulin. 

### 2.7. Histology and Immunohistochemistry

Nissl staining and immunohistochemical staining was performed as described previously [28,29]. Briefly, E18.5 mice were killed by decapitation and brains were fixed for 2 h in PBS containing 4% PFA. Thereafter, 25 µm transversal brain sections were generated by a Leica CM3050 S cryostat. For immunohistochemistry, brain sections were incubated for 1 h with 2% BSA, 3% goat serum, 10% donkey serum, and 0.5% NP40 in PBS and stained overnight at 4 °C with following primary antibody in 2% BSA and 0.5% NP40 in PBS: rabbit anti-neurofilament 200 (1:80, Sigma-Aldrich, St. Louis, MO, USA). As secondary antibody, AlexaFluor488 anti-rabbit IgG was used. For Nissl staining, brain sections were incubated in staining solution according to the manufacturer’s instructions. Image acquisition was done with a Leica TCS SP5 II confocal microscope setup and Leica M80 equipped with a Leica DFC295 camera (Leica Microsystems, Wetzlar, Germany).

### 2.8. Statistical Analysis 

Statistical tests were done in R or SigmaPlot. For comparing mean values between groups, Student’s *t*-test was performed. Analyzing the rescue conditions and protein expression over time and in different brain areas, ANOVA with post-hoc test (pairwise *t*-test with correction for multiple testing) was used. Stage distribution was tested for differences with χ^2^-test.

## 3. Results

### 3.1. CAP2 Is Expressed during Neuron Differentiation and Abundant in Growth Cones

**Figure 1:**
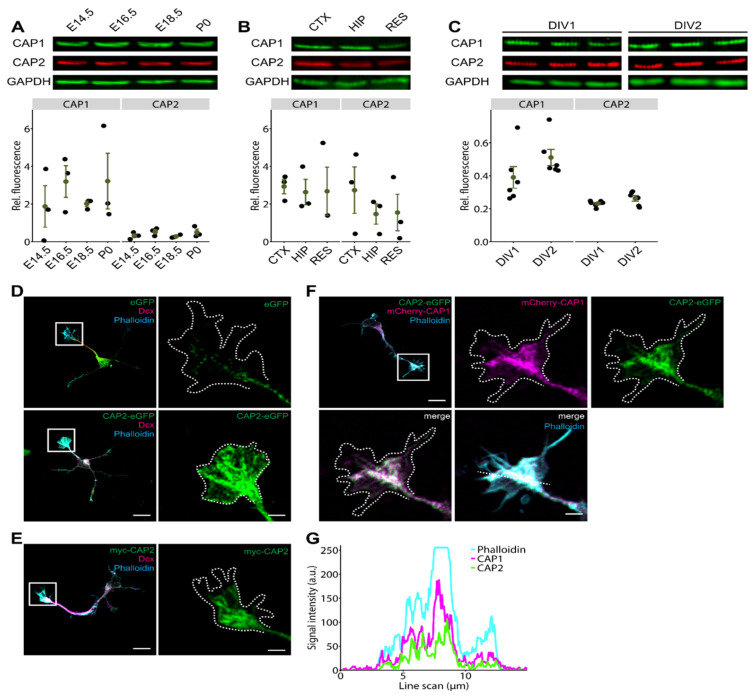
CAP2 is expressed in the embryonic brain and abundant in growth cones. (**A**) Immunoblots showing presence of CAP1 and CAP2 in cerebral cortex lysates during embryonic development, from embryonic day (E) 14.5 to postnatal day (P) 0. GAPDH was used as the loading control. Graph showing CAP1 and CAP2 levels normalized to the loading control, GAPDH. Black circles: normalized CAP1 and CAP2 levels in three biological replicates. Green circles and error bars: mean values (MV) and standard errors of the means (SEM). (**B**) Immunoblots showing expression levels of CAP1 and CAP2 in cerebral cortex (CTX) and hippocampus (HIP) lysates at P0. Additionally, lysate of residual brain (RES) was probed. GAPDH was used as the loading control. Graph showing CAP1 and CAP2 levels normalized to the loading control, GAPDH. Black circles: normalized CAP1 and CAP2 levels in three biological replicates. Green circles and error bars: MV and SEM. (**C**) Immunoblots showing presence of CAP1 and CAP2 in lysates from cultured cortical neurons (three biological replicates) after one and two days in vitro (DIV). GAPDH was used as the loading control. Graph showing CAP1 and CAP2 levels normalized to the loading control, GAPDH. Black circles: normalized CAP1 and CAP2 levels in six biological replicates. Green circles and error bars: MV and SEM. (**D**) Micrographs of hippocampal neurons at DIV1 expressing either GFP or GFP-CAP2 (green). Neurons were counterstained with the F-actin dye phalloidin (cyan) and the microtubule-associated protein doublecortin (Dcx, magenta). Boxes indicate areas shown at higher magnification, dashed lines outline growth cones as deduced from phalloidin staining. (**E**) Micrograph of a hippocampal neuron at DIV1 expressing myc-CAP2 visualized by myc antibody staining (green). Neuron was counterstained with phalloidin (cyan) and Dcx (magenta). Box indicates area shown at higher magnification, dashed line outlines growth cone. (**F**) Micrograph of a hippocampal neuron at DIV1 expressing GFP-CAP2 (green) and mCherry-CAP1 (magenta). Neuron was counterstained with phalloidin (cyan). Box indicates area shown at higher magnification, dashed line outlines growth cone. (**G**) Fluorescence intensity profile of GFP-CAP2, mCherry-CAP1 and phalloidin along white line shown in merge in G. Scale bars (in µm): 10 (low magnifications in (**D**–**F**)), 2 (high magnifications in (**D**–**F**)).

Previous immunoblot analysis revealed broad expression of CAP2 in the postnatal mouse brain including hippocampus [19]. However, it remains unknown whether CAP2 is present during embryonic brain development. We therefore performed immunoblots with mouse cerebral cortex lysates between embryonic day (E) 14.5 and postnatal day (P) 0. This analysis revealed the presence of CAP2 at all developmental stages examined, similar to CAP1 (Figure 1A). Quantification of signal intensities and normalization to GAPDH that was used as the loading control revealed constant expression levels for CAP1 and CAP2 during embryonic cerebral cortex lysates (Figure 1A; CAP1: E14.5: 1.87 ± 1.10, E16.5: 3.19 ± 0.84, E18.5: 2.01 ± 0.15, P0: 3.21 ± 1.48, *n* = 3, *p* = 0.52; CAP2: E14.5: 0.32 ± 0.11, E16.5: 0.53 ± 0.12, E18.5: 0.28 ± 0.05, P0: 0.50 ± 0.16, *n* = 3, *p* = 0.52). Immunoblot analysis further revealed CAP2 expression in hippocampal lysates from P0 mice (Figure 1B). A comparison between brain regions revealed similar expression levels for CAP1 and CAP2 in the cerebral cortex and hippocampus (CAP1: cortex: 2.93 ± 0.39, hippocampus: 2.63 ± 0.68, *n* = 3, *p* = 1; CAP2: cortex: 2.73 ± 1.23, hippocampus: 1.47 ± 0.54, *n* = 3, *p* = 0.92). Moreover, CAP2 was present in lysates from hippocampal neurons kept for one or two days in vitro (DIV; Figure 1C), and CAP2 expression levels were equal in DIV1 and DIV2 cultures, similar to CAP1 (CAP1: DIV1: 0.39 ± 0.07, DIV2: 0.51 ± 0.05, *n* = 6, *p* = 0.21; CAP2: DIV1: 0.23 ± 0.01, DIV2: 0.26 ± 0.02, *n* = 6, *p* = 0.95). Together, CAP2 was expressed in the embryonic and perinatal brain as well as in cultured hippocampal neurons, very similar to its close homolog CAP1 (Figure 1A–C) [9]. 

To determine the subcellular localization of CAP2 in differentiating neurons, we exploited hippocampal neurons isolated from E18.5 mice. Since CAP2-specific antibodies suitable for immunocytochemistry were not available, we introduced N-terminal GFP-tagged CAP2 (GFP-CAP2) into hippocampal neurons prior to plating by means of nucleofection. At DIV1, GFP-CAP2 was abundant in growth cones, which we identified by phalloidin staining of F-actin (Figure 1D). Unlike GFP-CAP2, fluorescence intensity in growth cones was much weaker in control neurons expressing GFP. In an independent experiment, we expressed CAP2 carrying a myc tag at its N-terminus (myc-CAP2). Similar to GFP-CAP2, myc-CAP2 was enriched in growth cones (Figure 1E). Moreover, we expressed GFP-CAP2 together with mCherry-tagged CAP1 in hippocampal neurons, and found both proteins enriched in growth cones (Figure 1F). Fluorescence intensity profiles of cross-sectional line scans revealed colocalization of GFP-CAP2, mCherry-CAP1, and phalloidin in growth cones (Figure 1G). Together, CAP2 was present during neuron differentiation and abundant in growth cones of differentiating neurons, very similar to CAP1. We therefore hypothesized a role for CAP2 in growth cone morphology and neuron differentiation.

#### 3.1.1. CAP2 Is Not Relevant for Early Neuron Differentiation

**Figure 2:**
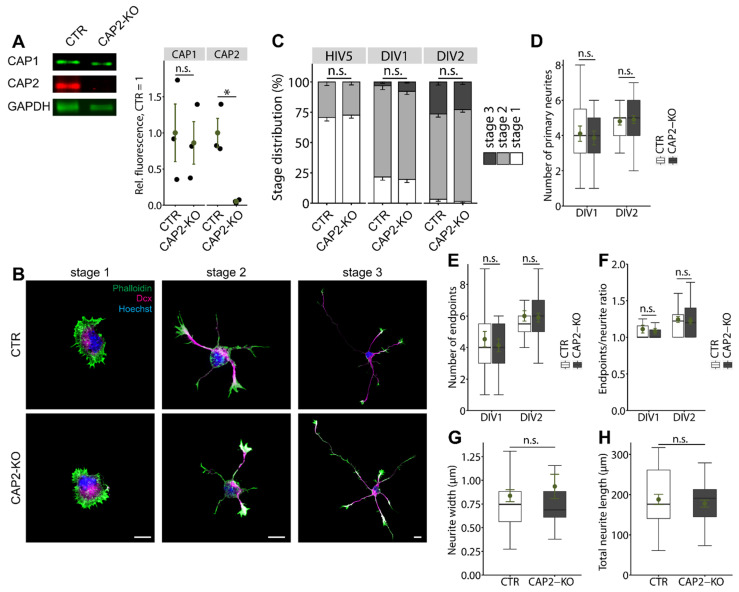
Normal differentiation and morphology of CAP2-KO neurons. (**A**) Immunoblots confirmed absence of CAP2 from CAP2-KO brain and unchanged expression levels of CAP1 in CAP2-KO brains. GAPDH was used as the loading control. Graph showing CAP1 and CAP2 levels normalized to the loading control, GAPDH, with CTR set to 1 in CTR and CAP2-KO brain lysates. Black circles: normalized CAP1 and CAP2 levels in three biological replicates. Green circles and error bars: MV and SEM. (**B**) Representative hippocampal neurons from CTR and CAP2-KO mice at differentiation stages 1 to 3 [31]. Neurons were stained with an antibody against doublecortin (Dcx, magenta), the DNA maker Hoechst (blue), and phalloidin (green). (**C**) Stage distribution for CTR and CAP2-KO neurons after five hours in vitro (HIV5) and at DIV1 and DIV2. Number of (**D**) primary neurites and (**E**) neurite endpoints in stage 2 CTR and CAP2-KO neurons at DIV1 and DIV2. (**F**) Endpoints/neurites ratio in stage 2 CTR and CAP2-KO neurons at DIV1 and DIV2. (**G**) Neurite width and (**H**) total neurite length in stage 2 CTR and CAP2-KO neurons. Scale bar (in µm): 10 (**B**). n.s.: *p* ≥ 0.05, * *p* < 0.05. Error bars represent SEM.

To test whether CAP2 was relevant for neuron differentiation, we analyzed hippocampal neurons isolated from CAP2-KO mice [12,30]. Immunoblots confirmed efficient CAP2 inactivation and revealed unchanged CAP1 expression levels in brain lysates from CAP2-KO mice (Figure 2A; CAP1: CTR: 1.00 ± 0.40, CAP2-KO: 0.86 ± 0.29, *n* = 3, *p* = 0.79; CAP2: CTR: 1.00 ± 0.20, CAP2-KO: 0.05 ± 0.01, *n* = 3, *p* < 0.05). To test whether CAP2 was relevant for neuron differentiation, we isolated hippocampal neurons from E18.5 CAP2-KO and compared them to neurons isolated from CAP2^+/+^ littermates that served as controls (CTR). First, we stained neurons at various time points after plating with an antibody against the neurite marker doublecortin (Dcx, Figure 2B). This allowed us to categorize neurons according to their differentiation stage, similar to previous studies [31]. After five hours in vitro (HIV5), we found the majority of CTR and CAP2-KO neurons in stage 1, i.e., they formed lamellipodia, but not yet neurites (Figure 2C; (%) CTR: 70.74 ± 2.93, CAP2-KO: 72.61 ± 2.46, *n* > 300 neurons from three mice). All other neurons possessed minor neurites, but not yet an axon and, hence, were assigned to stage 2 ((%) CTR: 29.26 ± 2.93, CAP2-KO: 27.39 ± 2.46). The stage distribution was not different between CTR and CAP2-KO at HIV5 (*p* = 0.74). Likewise, the stage distribution was unchanged in CAP2-KO neurons at DIV1 and DIV2 (Figure 2C; (%) DIV1: CTR: stage 1: 20.70 ± 2.71, stage 2: 76.29 ± 3.27, stage 3: 3.01 ± 1.09, CAP2-KO: stage 1: 18.91 ± 2.59, stage 2: 73.19 ± 2.66, stage 3: 7.89 ± 0.86, *n* > 200/3, *p* = 0.10; DIV2: CTR: stage 1: 3.24 ± 1.69, stage 2: 69.50 ± 2.63, stage 3: 27.26 ± 2.58, CAP2-KO: stage 1: 1.48 ± 0.98, stage 2: 75.50 ± 2.20, stage 3: 23.02 ± 1.94, *n* > 130/3, *p* = 0.44). Together, stage distribution of CAP2-KO neurons was unchanged between HIV5 and DIV2. 

We further exploited Dcx-stained neurons to determine their morphology by counting the numbers of primary neurites and neurite endpoints and by calculating the ratio of neurite endpoints and primary neurites (endpoint/neurite ratio) that we used as a readout for neurite branching. None of these parameters were changed in stage 2 CAP2-KO neurons at DIV1 or DIV2 (Figure 2D–F; DIV1: neurites: CTR: 4.11 ± 0.43, CAP2-KO: 3.87 ± 0.39, *p* = 0.68; endpoints: CTR: 4.53 ± 0.49, CAP2-KO: 4.13 ± 0.40, *p* = 0.54; endpoint/neurite ratio: CTR: 1.11 ± 0.05, CAP2-KO: 1.08 ± 0.04, *p* = 0.68, *n* = 15/5; DIV2: neurites: CTR: 4.81 ± 0.21, CAP2-KO: 4.90 ± 0.22, *p* = 0.75; endpoints: CTR: 6.00 ± 0.33, CAP2-KO: 5.90 ± 0.28, *p* = 0.82; endpoint/neurite ratio: CTR: 1.24 ± 0.04, CAP2-KO: 1.22 ± 0.04, *p* = 0.72, *n* > 31/3). Moreover, we determined neurite width and total neurite length in stage 2 neurons, two parameters that we found altered in CAP1-KO neurons [9]. Instead, neither neurite width nor total neurite length was altered in CAP2-KO neurons (Figure 2G–H; width: CTR: 0.84 ± 0.06, CAP2-KO: 0.94 ± 0.13, *p* = 0.49, *n* > 35/3; total length: CTR: 188.07 ± 12.59, CAP2-KO: 177.84 ± 8.74, *p* = 0.51, *n* > 30/3). Together, the morphology of stage 2 CAP2-KO neurons was unchanged. From the normal stage distribution in CAP2-KO cultures and from the normal morphology of CAP2-KO neurons we concluded that CAP2 was dispensable for neurite differentiation.

#### 3.1.2. CAP2 Is Dispensable for Growth Cone Size, Morphology, and Motility 

**Figure 3:**
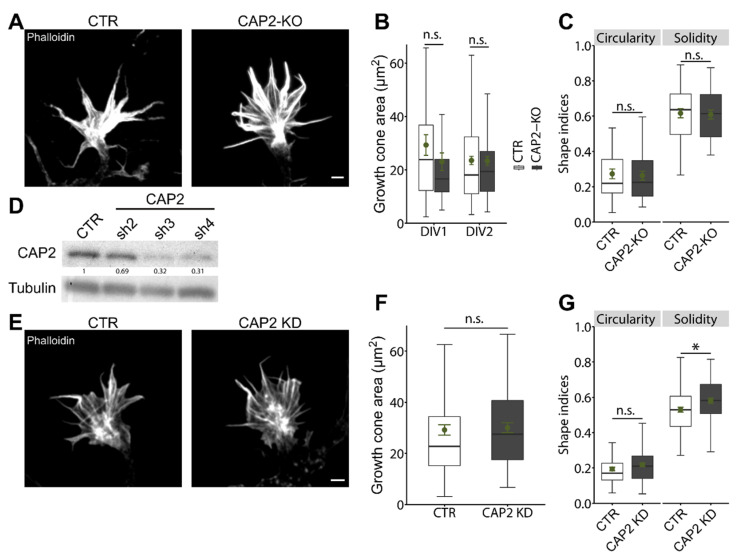
Normal growth cone size and morphology in CAP2-KO neurons. (**A**) Micrographs of phalloidin-stained growth cones from stage 2 CTR and CAP2-KO neurons. (**B**) Growth cone size in stage 2 CTR and CAP2-KO neurons at DIV1 and DIV2. (**C**) Growth cone shape indices for circularity and solidity for stage 2 CTR and CAP2-KO neurons. (**D**) Immunoblots with lysates from cerebral cortex neurons nucleofected with three different shRNAs directed against CAP2 (CAP2-sh2, CAP2-sh3, and CAP2-sh4) or with a control (CTR) shRNA. Tubulin was used as the loading control. Values indicate CAP2 levels in CAP2-KD neurons relative to CTR-shRNA-nucleofected controls. (**E**) Micrographs of phalloidin-stained growth cones from replated stage 2 neurons nucleofected with either CTR shRNA or a mixture of CAP2-sh3 and CAP2-sh4 (CAP2-KD). (**F**) Growth cone size and (**G**) shape indices for circularity and solidity in stage 2 neurons nucleofected with either CTR shRNA or CAP2-sh3/CAP2-sh4. Scale bars (in µm): 2 (**A**), 2 (**E**). * *p* < 0.05, n.s.: *p* ≥ 0.05. Error bars represent SEM.

The abundance of GFP-CAP2 in growth cones forced us to test whether CAP2 was relevant for growth cone size or morphology. To do so, we determined growth cone size in phalloidin-stained stage 2 neurons at HIV5 to DIV2. None of the investigated time points showed differences between CTR or CAP2-KO neurons (Figure 3A,B; (µm^2^) HIV5: CTR: 26.96 ± 1.56, CAP2-KO: 27.36 ± 1.96, *n* = 60/3, *p* = 0.87; DIV1: CTR: 29.32 ± 3.88, CAP2-KO: 23.08 ± 3.28, *n* = 30/3, *p* = 0.22; DIV2: CTR: 23.52 ± 1.57, CAP2-KO: 23.34 ± 1.60, *n* = 100/3, *p* = 0.93). Moreover, growth cone morphology was normal in CAP2-KO neurons as indicated by unchanged shape indices for circularity and solidity (Figure 3C; circularity: CTR: 0.27 ± 0.03, CAP2-KO: 0.26 ± 0.02, *p* = 0.78; solidity: CTR: 0.62 ± 0.03, CAP2-KO: 0.61 ± 0.03, *p* = 0.84, *n* = 32/3). Apart from CAP2-KO neurons, we tested whether acute CAP2 inactivation altered growth cone size or morphology in hippocampal neurons. To do so, we designed three different shRNAs directed against CAP2. Immunoblots confirmed efficient knockdown of CAP2 (CAP2-KD) in cerebral cortex neurons nucleofected with CAP2-sh3 and CAP2-sh4, while CAP2-sh2 only moderately decreased CAP2 levels when compared to control shRNA (CTR-shRNA)-nucleofected neurons (Figure 3D; sh2 0.69 ± 0.19, sh3: 0.32 ± 0.03, sh4: 0.31 ± 0.01, *n* = 3). To study growth cones, we nucleofected hippocampal neurons with a mixture of CAP2-sh3 and -sh4 prior to initial seeding, replated CAP2-KD neurons at DIV2 and analyzed growth cone size and morphology one day after replating (DAR1), similar to previous studies [9,25]. In DAR1 CAP2-KD neurons, growth cone size was not different from neurons nucleofected with CTR-shRNA (Figure 3E,F; (µm^2^) CTR: 29.17 ± 2.06, CAP2-KD: 30.04 ± 1.96, *n* = 60/3, *p* = 0.76). Likewise, the shape index circularity was not different between neurons of both groups, while the shape index solidity was slightly increased by 9% in CAP2-KD neurons (Figure 3G; circularity: CTR: 0.19 ± 0.01, CAP2-KD: 0.22 ± 0.01, *n* = 60/3, *p* = 0.12; solidity: CTR: 0.53 ± 0.01, CAP2-KD: 0.58 ± 0.01, *n* = 60/3, *p* < 0.05). Together, neither acute nor systemic CAP2 inactivation altered growth cone size. Moreover, growth cone morphology was unchanged in CAP2-KO neurons and only slightly altered upon acute CAP2 knockdown. From these data we concluded that CAP2 was largely dispensable in growth cones. Indeed, growth cones from CAP2-KO neurons were as motile as the ones from CTR neurons (Movies S1–S2).

#### 3.1.3. CAP2 Is Dispensable for Brain Development

**Figure 4:**
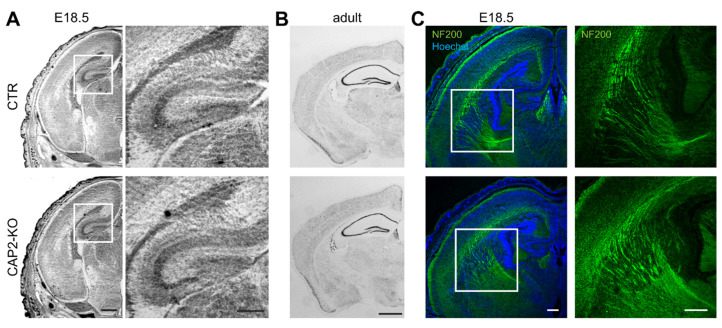
CAP2 inactivation does not cause obvious histological alterations in the mouse brain. (**A**) Nissl staining of transversal brain sections from CTR and CAP2-KO mice at E18.5. Boxes indicate area shown at higher magnification. (**B**) Nissl staining of transversal brain sections from adult CTR and CAP2-KO mice. (**C**) Antibody staining against the axon marker neurofilament (green) in transversal brain sections from E18.5 CTR and CAP2-KO mice. Sections were counterstained with DNA dye Hoechst (blue). Boxes indicate area shown at higher magnification. Scale bars (in µm): 500 (low magnification), 250 (high magnification) (**A**), 1000 (**B**), and 100 (**C**).

The data we presented so far revealed CAP2 expression in differentiating neurons and abundance in growth cones, similar to CAP1. However, while we found CAP1 to be relevant for neuron differentiation, growth cone morphology, and growth cone motility [9], none of these processes was impaired in CAP2-KO neurons. Impaired differentiation of CAP1-KO neurons was associated with hypomorphic fiber tracks in brains from CAP1-KO mice and a somewhat altered hippocampus morphology [9]. Such changes were not present in brains from CAP2-KO mice. Specifically, Nissl-stained transversal brains sections revealed no obvious differences in cerebral cortex or hippocampus anatomy between CTR and CAP2-KO mice at E18.5 or in adult mice (Figure 4A,B). Moreover, antibody staining against the axon marker neurofilament suggested a normal appearance of fiber tracks in CAP2-KO brains (Figure 4C), different from CAP1-KO mice [9]. Together, CAP2-KO mice did not display any gross defects in brain development.

#### 3.1.4. CAP2 Can Rescue Neuron Morphology and Differentiation in CAP1-KO Neurons 

**Figure 5:**
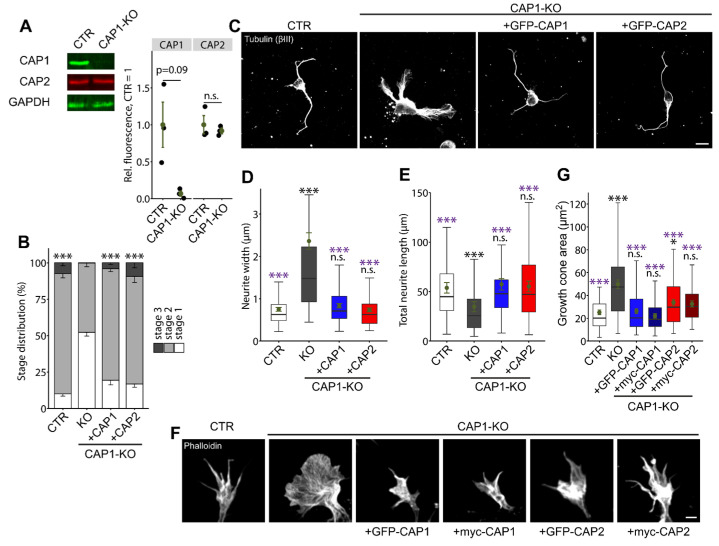
CAP2 overexpression partially rescues morphological changes in CAP1-KO neurons. (**A**) Immunoblots of three biological replicates showing absence of CAP1 from the CAP1-KO brain and unchanged expression of CAP2. GAPDH was used as the loading control. Graph showing CAP1 and CAP2 levels normalized to the loading control, GAPDH with CAP1 set to 1 in CAP1-KO brain lysates. Black circles: normalized CAP1 and CAP2 levels in three biological replicates. Green circles and error bars: MV and SEM. (**B**) Stage distribution of DIV1 CTR neurons, CAP1-KO neurons, and CAP1-KO neurons expressing either GFP-CAP1 or GFP-CAP2. (**C**) βIII-tubulin-stained neurons (CTR, CAP1-KO, and CAP1-KO expressing either GFP-CAP1 or GFP-CAP2) that were used for the analysis of neurite width and total neurite length shown in (**D**,**E**). (**F**) Growth cones from phalloidin-stained neurons (CTR, CAP1-KO, and CAP1-KO expressing either GFP- or myc-tagged CAP1 or GFP- or myc-tagged CAP2) that were used for the analysis of growth cones size shown in (**G**). Scale bars (in µm): 10 (**C**), 2 (**F**). n.s.: *p* ≥ 0.05, * *p* < 0.05, *** *p* < 0.001. Error bars represent SEM. Black asterisks and n.s. indicate comparison to CTR neurons and purple asterisks and n.s. indicate comparison to CAP1-KO neurons.

From our data and our previous analysis in CAP1-KO neurons we concluded that CAP2 is dispensable for neuron differentiation and brain development and that CAP1 is the limiting factor in differentiating neurons [9]. Absence of any defects in CAP2-KO neurons could be explained by functional redundancy of CAP1 and CAP2 during neuron differentiation. To determine whether both CAPs share redundant functions, we tested whether overexpression of CAP2 can rescue morphological changes in CAP1-deficient neurons. Hippocampal neurons deficient for CAP1 were isolated from brain-specific CAP1-KO mice that we achieved by crossing a conditional CAP1 strain and Nestin-Cre transgenic mice (CAP1^flx/flx,Nestin-Cre^) [9,23]. Immunoblots confirmed absence of CAP1 from hippocampal lysates obtained from E18.5 CAP1^flx/flx,Nestin-Cre^ mice (termed CAP1-KO), CAP1^flx/flx^ littermates served as controls (CTR). Compared to CTR, CAP2 expression levels were unchanged in the CAP1-KO hippocampus (Figure 5A; CAP1: CTR: 1.00 ± 0.31, CAP1-KO: 0.07 ± 0.04, *n* = 3, *p* = 0.09; CAP2: CTR: 1.00 ± 0.12, CAP1-KO: 0.92 ± 0.04, *n* = 3, *p* = 0.58). Similarly to our previous study [9], differentiation was impaired in the CAP1-KO neuron. Specifically, the fraction of stage 1 neurons was increased on the expense of stage 2 and stage 3 neurons at DIV1 (Figure 5B; (%) CTR: stage 1: 10.10 ± 1.61, stage 2: 82.41 ± 2.84, stage 3: 7.49 ± 2.04; CAP1-KO: stage 1: 52.27 ± 2.59, stage 2: 47.47 ± 2.44, stage 3: 0.27 ± 0.28, *n* > 300/3, *p* < 0.001). Moreover, stage 2 CAP1-KO displayed an increased neurite width and a reduced total neurite length (Figure 5C–E; (µm) width: CTR: 0.75 ± 0.04, CAP1-KO: 2.36 ± 0.19, *p* < 0.001, *n* > 80/3; length: CTR: 53.82 ± 5.38, CAP1-KO: 34.72 ± 5.14, *p* < 0.05, *n* < 40/3). As expected, expression of *N*-terminal GFP-tagged CAP1 (GFP-CAP1) normalized stage distribution in CAP1-KO neurons (stage 1: 19.39 ± 3.19, stage 2: 76.53 ± 2.01, stage 3: 4.08XY ± 1.25, *n* > 90/3, *p* < 0.001), and it reduced neurite width and increased total neurite length ((µm) width: 0.84 ± 0.05, *p* < 0.001, *n* = 85/3; length: 57.40 ± 5.82, *p* < 0.05, *n* > 40/3). Similarly, overexpression of GFP-CAP2 normalized stage distribution in CAP1-KO neurons (Figure 5B; stage 1: 16.82 ± 2.26, stage 2: 73.83 ± 4.17, stage 3: 9.35 ± 3.40, *n* > 100/3, *p* < 0.001). Moreover, GFP-CAP2 overexpression rescued neurite width and total neurite length (Figure 5C–E; (µm) width: 0.74 ± 0.04, *p* < 0.001, *n* > 90/3; length: 55.22 ± 5.46, *p* < 0.05, *n* > 35/3). Next, by determining growth cone size we tested whether CAP2 overexpression can compensate for CAP1 loss in growth cones. As reported before [9], growth cones were enlarged by 98% in CAP1-KO neurons (Figure 5F,G; (µm^2^) CTR: 25.14 ± 2.07, CAP1-KO: 49.86 ± 3.71, *n* = 60/3, *p* < 0.001). Growth cone size in CAP1-KO neurons was reduced by expression of either N-terminal myc-tagged CAP1 (myc-CAP1) or GFP-CAP1 ((µm^2^): myc-CAP1: 22.08 ± 1.97, *p* < 0.001, *n* = 42/3; GFP-CAP1: 26.26 ± 2.10, *p* < 0.001, *n* = 80/3), and growth cone size in these neurons was not different from CTR neurons (myc-CAP1: *p* = 0.49; GFP-CAP1: *p* = 0.74). Similar to myc-CAP1 and GFP-CAP1, overexpression of either GFP-CAP2 or myc-CAP2 reduced growth cone size in CAP1-KO neurons ((µm^2^) myc-CAP2: 32.54 ± 2.63, *p* < 0.001, *n* = 37/3; GFP-CAP2: 33.92 ± 2.44, *p* < 0.001, *n* = 60/3). When compared to CTR neurons, growth cone size was not different in myc-CAP2-overexpressing CAP1-KO neurons (*p* = 0.10), but remained enlarged in GFP-CAP2-overexpressing CAP1-KO neurons (*p* < 0.05). Together, CAP2 overexpression reduced growth cone size in CAP1-KO neurons, suggesting redundant functions of CAP1 and CAP2 in growth cones.

## 4. Discussion

The present study aimed at deciphering the role for CAP2 in early neuron differentiation, which we found expressed in the embryonic and perinatal brain and abundant in growth cones from hippocampal neurons, similar to its close homolog CAP1 [9,11,32,33]. While we recently reported important CAP1 functions in neuron differentiation, growth cone morphology, and neuron connectivity in the mouse brain [9], neuron differentiation and growth cone morphology was normal in CAP2-KO neurons, and CAP2-KO mice did not display obvious defects in brain development or neuron connectivity. Interestingly, CAP2 overexpression rescued morphological changes in isolated hippocampal neurons from CAP1-KO mice, suggesting that CAP1 and CAP2 share redundant functions in differentiating neurons. 

Normal stage distribution and morphology of isolated hippocampal CAP2-KO neurons together with normal growth cone size and morphology and absence of any obvious histological changes in CAP2-KO brains revealed that CAP2 was dispensable for neuron differentiation and brain development. Instead, a previous study unraveled important functions for CAP2 in differentiated neurons [19]. Specifically, this study implicated CAP2 in regulating the morphology of dendritic spines, the F-actin-enriched postsynaptic compartments of most excitatory synapses in the brain. Moreover, it revealed an interaction of CAP2 with the actin-depolymerizing protein cofilin1, a key regulator of synaptic actin dynamics, spine morphology, synaptic plasticity, brain function, and behavior [21,34,35,36,37,38,39,40]. Further, this study implicated the interaction of CAP2 with cofilin1 in structural plasticity of excitatory synapses, and it provided evidence that CAP2-cofilin1 interaction is compromised in Alzheimer’s disease patients, which may contribute to the underlying disease mechanism [19]. Spine morphological changes upon CAP2 inactivation were also reported in a second study, which unfortunately did not provide detailed mechanistic insights [41]. Together, these studies and our findings in CAP2-KO neurons and brains suggest that CAP2 acquires important functions in differentiated neurons, but is dispensable during neuron differentiation.

Unlike most other cell types, hippocampal neurons express both CAP family members, and our group recently demonstrated important functions for CAP1 in regulating the actin cytoskeleton during neuron differentiation [9]. Specifically, we showed that CAP1 controls organization and dynamics of F-actin in growth cones. F-actin defects impaired growth cone function in CAP1-KO neurons and retarded their differentiation, which likely caused compromised neuron connectivity in CAP1-KO mouse brains [9]. Interestingly, we found a cooperation of CAP1 and cofilin1 during neuron differentiation, and our data suggested functional interdependency for both actin regulators in growth cones. Hence, CAP1 was relevant for cofilin1-dependent actin dynamics in growth cones of differentiating neurons, while CAP2 controls cofilin1 in dendritic spines from differentiated neurons.

Absence of any defects in differentiating CAP2-KO neurons led us suggest that CAP1 is the dominant family member during neuron differentiation and brain development. However, CAP2 overexpression rescued neuron differentiation as well as neurite width and total neurite length in CAP1-KO neurons, and it partially rescued growth cone size. These data revealed that CAP2 can compensate CAP1 inactivation in isolated neurons during differentiation, suggesting that CAP1 and CAP2 share overlapping and redundant functions during neuron differentiation. Moreover, this finding led us to speculate compensatory mechanisms in single KO neurons, which could explain normal differentiation and growth cone morphology in CAP2-KO neurons. Differently from the brain, CAP1 is absent from skeletal muscles and only weakly expressed in the heart [10,11]. Hence, CAP1 cannot compensate CAP2 inactivation in striated muscles, thereby explaining why systemic CAP2-KO mice displayed defects in heart physiology and myofibril differentiation, but not in neuron differentiation or brain development [12,14,15]. Functional redundancy of mouse CAP1 and CAP2 has been postulated earlier [15], and is in good agreement with the roughly 60% identity and 75% similarity both proteins share in their primary sequences [1,11]. Moreover, the overlapping functions in the brain is supported by recent findings (i) demonstrating that CAP1 and CAP2 both interact with cofilin1 to control neuronal actin dynamics [9,19] and (ii) identifying both CAP1 and CAP2 as components of a protein complex isolated from the mouse brain that was able to inhibit the F-actin assembly factor INF2 [6,8]. Apart from this, most studies that investigated the molecular functions of mammalian CAPs primarily focused on CAP1 (for review: [2]), and it therefore remains to be tested whether (i) CAP1 and CAP2 acquired specific functions in actin regulation, (ii) they possess specific interaction partners, (iii) they are controlled by specific regulatory mechanisms, (iv) they are addressed by specific signaling cascades, and (v) they share redundant functions in vivo. It will be exciting to decipher, in future studies, common and specific upstream and downstream mechanisms of CAP1 and CAP2 in neurons.

## Data Availability

Data sharing not applicable.

## Supplementary Materials

The following are available online at https://www.mdpi.com/article/10.3390/cells10061525/s1, Movie S1: Motility of a growth cone from a CTR neuron. Movie showing a CTR growth cone imaged by differential interference contrast (DIC) microscopy. Many protruding and retracting filopodia were present during image acquisition of 10 min. Scale bar: 2 µm. Movie S2: Motility of a growth cone from a CAP2-KO neuron. DIC-imaged growth cone from CAP2-KO neuron displaying filopodia protrusions and retractions. Scale bar: 2 µm.
